# Peripheral Immune Cell Profiles and Circulating Cytokine Signals in Vietnamese Children Hospitalized with Acute Asthma Exacerbations: A Cross-Sectional Study

**DOI:** 10.3390/pediatric18050135

**Published:** 2026-10-09

**Authors:** Long Hoang Do, Xuyen Thi Bao Nguyen, Cua Thi Hong Trinh, Truc Thi Huong Dinh, Dung Ngoc Tran, Tu Thi Cam Le, Thang Nguyen

**Affiliations:** 1 Department of Pathophysiology–Immunology, Can Tho University of Medicine and Pharmacy, 179 Nguyen Van Cu Street, Tan An Ward, Can Tho City 90000, Vietnam; tthcua@ctump.edu.vn (C.T.H.T.); dthtruc@ctump.edu.vn (T.T.H.D.); tranngocdung@ctump.edu.vn (D.N.T.); 2 Department of Physiology, Pathophysiology and Immunology, Faculty of Medicine, School of Medicine and Pharmacy, Tra Vinh University, 126 Nguyen Thien Thanh Street, Hoa Thuan Ward 87118, Vinh Long Province, Vietnam; ntbxuyen@tvu.edu.vn; 3 Department of Pharmacology and Clinical Pharmacy, Can Tho University of Medicine and Pharmacy, Can Tho City 90000, Vietnam; ltctu@ctump.edu.vn (T.T.C.L.); nthang@ctump.edu.vn (T.N.)

**Keywords:** acute asthma exacerbation, children, cytokines, eosinophils, Vietnam

## Abstract

Background/Objectives: We characterized peripheral inflammatory patterns during pediatric acute asthma exacerbations and explored clinical and cytokine differences by blood eosinophil status. Methods: This cross-sectional study included 59 children aged ≥5 years hospitalized in Can Tho, Vietnam, during April 2023–July 2024. Blood counts and six serum cytokines were classified using prespecified study thresholds. Associations were reassessed using Pearson or exact tests, crude odds ratios, and Holm adjustment across 46 unique exploratory hypotheses. Results: Study-defined leukocytosis, eosinophilia, neutrophilia, and basophilia occurred in 71.2%, 62.7%, 54.2%, and 22.0%, respectively. IL-6, IL-5, and TNF-α met their study thresholds in 69.5%, 23.7%, and 3.4%; IL-4, IL-12p70, and IFN-γ remained below those thresholds. The eosinophilia–severity comparison was not significant (exact *p* = 0.368). Personal and family allergy associations did not remain significant after multiplicity adjustment (adjusted *p* = 0.348 and 0.429). Cytokine-threshold frequencies did not differ significantly between eosinophil strata. Conclusions: Peripheral inflammatory patterns were heterogeneous, but no tested association survived multiplicity adjustment. These descriptive findings do not validate blood-based airway phenotypes or severity prediction. Larger longitudinal studies should standardize treatment and sampling-time records, infection assessment, and assay limits.

## 1. Introduction

Asthma is a heterogeneous chronic airway disease characterized by variable respiratory symptoms and airflow limitation [1]. In children, acute exacerbations contribute to emergency visits, hospitalization, interrupted schooling, and family burden. An accessible description of inflammation during an exacerbation could help define questions for subsequent studies of prognosis and treatment response; it is not, by itself, evidence of a clinically validated phenotype.

The immunopathology of asthma involves structural airway cells, eosinophils, neutrophils, basophils, and interacting T-helper-cell pathways [2,3]. Clinical phenotypes do not necessarily correspond to a single molecular endotype, particularly in childhood and severe disease [4,5,6,7]. Blood eosinophils are among the better-studied peripheral markers, but their performance as surrogates for airway inflammation and future disease burden depends on the population, clinical state, and measurement conditions [8,9,10,11]. Serum cytokines offer complementary biological information, although a binary assay classification cannot establish the presence or absence of an inflammatory pathway.

A Vietnamese study previously described leukocyte and cytokine profiles during pediatric acute asthma exacerbations [12]. Further observations from the Mekong Delta may help characterize the range of peripheral inflammatory findings encountered in routine hospital care. The primary objective of the present study was to characterize peripheral inflammatory patterns, defined by study-threshold blood-cell and cytokine profiles, in children hospitalized with acute asthma exacerbations. The secondary objective was to explore associations with clinical characteristics. During revision, we additionally compared clinical features and cytokine-threshold frequencies between children with blood eosinophil counts ≥300 and <300 cells/µL. These blood-defined groups were not intended to establish airway inflammatory phenotypes or molecular endotypes.

## 2. Materials and Methods

### 2.1. Study Design and Setting

This analytical cross-sectional study was conducted at Can Tho Children’s Hospital, Vietnam, from April 2023 to July 2024. There was no non-asthmatic control group or paired assessment during stable asthma or recovery.

### 2.2. Participants and Sample Size

**Figure 1:**
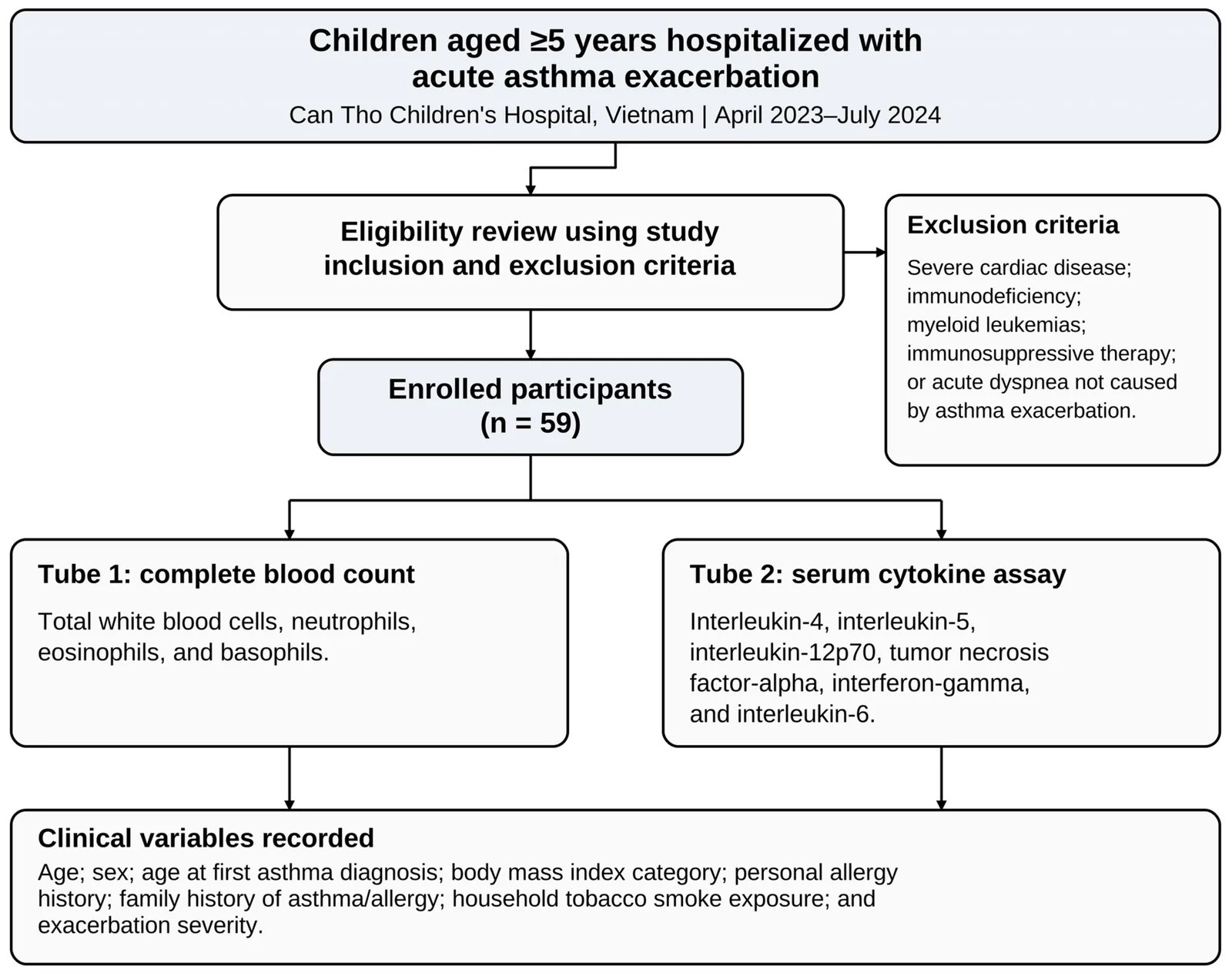
Study workflow, eligibility assessment, and laboratory measurements. The side branch lists exclusion criteria; screening and exclusion counts were not available. The enrolled sample comprised 59 children. IL-12p70 is the IL-12 analyte measured by the assay panel.

Children aged ≥5 years were eligible when diagnosed with asthma according to the Global Initiative for Asthma criteria used in the protocol and evaluated during an acute exacerbation [1]. The cohort included children with a previously documented diagnosis and children first diagnosed at the enrollment admission. Exclusion criteria were severe cardiac disease, immunodeficiency, myeloid leukemias, use of immunosuppressive drugs such as cyclosporine, azathioprine, methotrexate, or cyclophosphamide, and acute dyspnea due to causes other than asthma. These exclusion criteria did not establish that participants were corticosteroid-naive. Convenience sampling was used, and 59 children were enrolled (Figure 1).

The sample size was calculated to estimate a single proportion using *n* = Z^2^*p*(1 − *p*)/d^2^, with Z = 1.96 for a two-sided 95% confidence level, an expected leukocytosis proportion *p* = 0.82 based on the 2017 thesis by Le Thi Thu Huong, and absolute precision d = 0.10 [13]. The calculated value was 56.70, rounded upward to a minimum of 57; 59 children were enrolled. This calculation was not designed to establish power for secondary association or subgroup analyses.

### 2.3. Clinical Variables

Clinical variables were age, sex, age at first asthma diagnosis (<2, 2–5, or >5 years), protocol body mass index (BMI)-for-age category, personal allergic history, family history of asthma/allergy, household tobacco smoke exposure, and exacerbation severity. The original protocol grouped BMI-for-age z-scores as <−2, −2 to +2, and >+2 using WHO charts [14]. These operational categories are reported without redefining the original data or presenting them as the complete WHO overweight/obesity classification. Personal allergic history included documented allergic rhinitis, eczema, urticaria, allergic conjunctivitis, or known allergen sensitivity. Family history was positive when asthma or allergic disease was reported in parents, grandparents, or siblings. Severity was categorized as mild, moderate, or severe using the Pediatric Asthma Score specified in the protocol.

Microbiological testing, including testing for *Mycoplasma pneumoniae*, was not performed. Medication histories before admission and before blood sampling, including systemic corticosteroid dose, route, and timing, were not collected systematically. Consequently, the number of pretreated children could not be established reliably and treatment-adjusted or treatment-stratified analyses were not performed.

### 2.4. Blood Collection and Laboratory Procedures

Blood was collected immediately following admission; patient-specific admission-to-draw intervals were unavailable for the present analysis. A 4-mL peripheral venous sample was collected from each child. One tube was used for a complete blood count on an automated laser hematology analyzer at Can Tho Children’s Hospital. A second tube without anticoagulant was used for serum cytokine measurement. The processing protocol specified serum separation within 6 h, approximately 30 min for clotting, two centrifugations at 5000 rpm for 15 min, aliquoting into 1.5-mL tubes, storage at −80 °C, transport with dry ice within 24 h, and a single thaw before analysis.

Serum cytokines were measured using the ProcartaPlex Human Essential Th1/Th2 Cytokine Panel, 6plex (Thermo Fisher Scientific, Waltham, MA, USA, EPX060-10009-901), with Luminex technology on a Bio-Plex platform (Bio-Rad, Hercules, CA, USA) at the Department of Immunology, Military Medical University, Hanoi. The six analytes were IL-4, IL-5, IL-6, IL-12p70, IFN-γ, and TNF-α. The available laboratory summary described weighted five-parameter logistic standard curves and reported R^2^ values of 0.9998 for IL-4, 0.9989 for IL-5, 0.9978 for IL-6, and 0.9973 for IFN-γ. Original run-level reports were unavailable for independent reassessment. These fit statistics alone do not establish reliable quantification at low concentrations or exclude pre-analytical effects.

### 2.5. Operational Biomarker Definitions

The protocol classified total white blood cells ≥10,000 cells/µL, neutrophils ≥7000 cells/µL, eosinophils ≥300 cells/µL, and basophils ≥50 cells/µL as increased; cells/µL and cells/mm^3^ are equivalent units. Nguyen Cong Khanh’s pediatric hematology text was the protocol reference [15]. These thresholds are reported as study-defined operational cutoffs, not externally validated age-specific pediatric reference limits. Individual absolute counts were unavailable for the revision-stage analyses, precluding reclassification using alternative age-specific limits.

Cytokine classification thresholds were IL-4 ≥ 10.62, IL-5 ≥ 6.836, IL-12p70 ≥ 7.642, TNF-α ≥ 6.152, IFN-γ ≥ 14.233, and IL-6 ≥ 9.473 pg/mL. The available analytical documentation did not establish that these values represented the lot-specific limits of detection (LODs), lower limits of quantification (LLOQs), or clinical reference limits. Manufacturer guidance notes that quantification ranges can vary between lots [16]. Accordingly, results are reported as “at or above the study threshold” or “below the study threshold.” A below-threshold classification does not indicate a zero concentration, analytical non-detection, or absence of the corresponding inflammatory pathway.

### 2.6. Statistical Analysis

The original analyses used SPSS version 20.0. During revision, frequencies, proportions, and contingency-table analyses were recalculated from aggregate tables using Python 3.13.5, SciPy 1.17.0, and statsmodels 0.14.6. Exact probabilities, ORs, confidence intervals, and Holm-adjusted *p*-values were checked with a second implementation. No patient-level observations were reconstructed or imputed. Age is reported as the original mean ± standard deviation because individual ages were unavailable for recalculation. All proportions are accompanied by their denominators.

Pearson χ^2^ tests without continuity correction were used when all expected cell counts were at least five. Otherwise, two-sided Fisher exact tests were used for 2 × 2 tables and Fisher–Freeman–Halton exact tests for larger tables. Exact *p*-values were obtained by exhaustive enumeration of fixed-margin tables, summing probabilities no larger than the observed-table probability; no Monte Carlo approximation was used. Unadjusted odds ratios (ORs) were computed as ad/bc with 95% log-Wald confidence intervals when all four cells were nonzero. These approximate intervals are descriptive and can be imprecise with small cells. Where a zero cell prevented a finite log-Wald estimate, results are labeled not estimable (NE); no continuity correction was added. For factors with more than two categories, the *p*-value is the global test, whereas ORs compare individual categories with the stated reference.

All associations are exploratory. Holm adjustment was applied to a single revision-stage family of 46 unique global hypotheses [17]: 42 original comparisons (six clinical factors × seven varying biomarkers), plus four additional comparisons requested during review (sex versus eosinophil stratum, and eosinophil stratum versus IL-5, TNF-α, and IL-6 threshold status). Clinical comparisons repeated in the eosinophil-stratum table were counted only once. No test was performed for IL-4, IL-12p70, or IFN-γ because all participants had the same below-threshold classification. Both raw and adjusted *p*-values are reported; adjusted *p* < 0.05 was the revision-stage significance criterion. Confidence intervals were not multiplicity-adjusted. No multivariable model, continuous-cytokine analysis, or joint eosinophilic/neutrophilic phenotype classification was inferred from aggregate data.

Generative AI assistance was used during revision for language editing, response drafting, preparation of aggregate-table verification code, and figure typesetting. It was not used to generate or impute participant observations. Details are provided in the Acknowledgments.

### 2.7. Ethical Considerations

The study was conducted in accordance with the Declaration of Helsinki and approved by the Ethics Committee in Biomedical Research of Can Tho University of Medicine and Pharmacy on 20 March 2023 (approval No. 23.031.HV/PCT-HĐĐĐ). Informed consent was obtained from parents or legal guardians, and participating children agreed to take part before enrollment.

## 3. Results

### 3.1. Study Population

The 59 children had a mean age of 7.75 ± 2.7 years; 42 (71.2%) were boys. Forty-five children (76.3%) had a previous asthma diagnosis, and 14 (23.7%) were first diagnosed at enrollment. Exacerbations were mild in 22 children (37.3%), moderate in 33 (55.9%), and severe in four (6.8%) (Table 1).

### 3.2. Peripheral Blood and Cytokine Patterns

Study-defined increases were recorded for total white blood cells in 42/59 (71.2%), eosinophils in 37/59 (62.7%), neutrophils in 32/59 (54.2%), and basophils in 13/59 (22.0%). IL-6 met its study threshold in 41/59 (69.5%), IL-5 in 14/59 (23.7%), and TNF-α in 2/59 (3.4%). No sample reached the respective IL-4, IL-12p70, or IFN-γ study threshold (Table 2). These binary data do not distinguish measurable low concentrations from values below an assay-specific LOD or LLOQ.

### 3.3. Clinical Comparisons by Eosinophil Stratum

Thirty-seven children had eosinophil counts ≥300 cells/µL and 22 had counts <300 cells/µL. Eosinophilia was present in 14/22 children with mild, 19/33 with moderate, and 4/4 with severe exacerbations. The global two-sided exact test did not show a statistically significant association with exacerbation severity (*p* = 0.368).

The crude OR for eosinophilia was 4.49 (95% CI 1.45–13.97; raw *p* = 0.008) for personal allergic history and 4.38 (95% CI 1.39–13.83; raw *p* = 0.010) for family history of asthma/allergy. The corresponding Holm-adjusted *p*-values were 0.348 and 0.429. No clinical comparison remained statistically significant after multiplicity adjustment (Table 3).

### 3.4. Cytokines by Eosinophil Stratum and Other Associations

The proportions meeting the IL-5 threshold were 7/22 (31.8%) in the <300 cells/µL group and 7/37 (18.9%) in the ≥300 cells/µL group (raw *p* = 0.260). The corresponding IL-6 proportions were 14/22 (63.6%) and 27/37 (73.0%) (raw *p* = 0.451). Both TNF-α threshold-positive observations occurred in the ≥300 cells/µL group (two-sided Fisher *p* = 0.524). All adjusted *p*-values for these comparisons were 1.000 (Table 4). No inference about equivalence between groups is intended.

Detailed counts, global tests, reference categories, and crude ORs for all six original clinical factors and seven varying biomarkers are presented in Supplementary Tables S1–S6. Supplementary Table S7 identifies the four new, nonduplicated hypotheses. None of the 46 unique comparisons reached the Holm-adjusted 0.05 criterion.

## 4. Discussion

This hospital-based cohort showed frequent study-defined increases in total white blood cells and eosinophils, with IL-6 most often meeting its cytokine threshold. None of the evaluated associations remained statistically significant after multiplicity adjustment. The eosinophilia–severity comparison was not significant (two-sided exact *p* = 0.368), and the four severe cases do not support a precise estimate of severity-related differences. The findings therefore describe peripheral inflammatory patterns and generate hypotheses rather than validate a clinical biomarker.

The previous Vietnamese study by Nguyen-Thi-Dieu and colleagues included 127 children during acute exacerbations and 30 healthy controls; the mean age of the exacerbation group was 58 ± 38 months [12]. It reported leukocytosis in 103/127 (81.1%), neutrophilia in 84/127 (66.1%), and eosinophilia in 38/122 (31.1%). Corresponding proportions in our older, uncontrolled cohort were 42/59 (71.2%), 32/59 (54.2%), and 37/59 (62.7%). Severe exacerbations comprised 63/127 (49.6%) in that study and 4/59 (6.8%) here. Differences in age, severity distribution, thresholds, and measurement methods preclude attributing these contrasts to regional biology. Cytokine concentrations reported as continuous measurements in the earlier study cannot be equated directly with our threshold frequencies.

The predominance of boys is consistent with the importance of age and sex in childhood asthma epidemiology, but this sample cannot evaluate sex-specific mechanisms [18]. Personal and family allergic histories showed unadjusted associations with eosinophil status, although these did not meet the multiplicity-adjusted criterion. Pediatric evidence linking blood eosinophils to subsequent exacerbations, together with evidence of temporal variability in adults with severe eosinophilic asthma, provides a rationale for longitudinal research rather than an explanation proven by this cross-sectional sample [19,20]. The ≥300 and <300 cells/µL strata are peripheral blood categories; the latter cannot automatically be called neutrophilic asthma. The aggregate tables do not establish the joint distribution of eosinophilia and neutrophilia.

Innate immune activation and neutrophilic inflammation are relevant to acute asthma, while circulating IL-6 may reflect several inflammatory and metabolic processes [21,22,23]. Our observations cannot discriminate these explanations. Infection is a particularly important unmeasured confounder. The local study by Bui and colleagues and related work on *Mycoplasma pneumoniae* place infection within the pediatric asthma differential [24,25,26]. Because we performed no microbiological testing, neither the IL-6 classifications nor neutrophil counts can be attributed specifically to asthma biology or used to infer an infecting pathogen.

The absence of samples meeting the IL-4, IL-12p70, and IFN-γ study thresholds should not be interpreted as biological absence or suppression of the corresponding pathways. Peripheral blood measurements may differ from inflammatory activity at the airway. Pre-analytical handling, assay sensitivity, and treatment before sampling remain alternative explanations. The processing protocol and standard-curve R^2^ values do not exclude analyte degradation or establish reliable quantification at low concentrations. Lot-specific LOD/LLOQ documentation and original concentration/readout files were unavailable for reassessment, limiting interpretation of below-threshold results. Although IL-5-targeted therapies in pediatric allergic disease and other biologics in adult severe asthma have been studied, these binary data do not establish treatment eligibility or predict a biologic response [27,28].

Household tobacco smoke and adiposity remain clinically important in asthma, despite the absence of multiplicity-adjusted associations in this sample [29,30,31,32]. The exposure measure was binary, and the protocol BMI categories were coarse. Null results from these small strata should not be interpreted as evidence that smoke exposure or metabolic factors are unimportant.

The clinical relevance for Vietnam lies in defining what can—and cannot—be learned from routinely accessible blood counts in a small pediatric hospital cohort. A complete blood count can describe a child’s peripheral inflammatory state, but this study does not demonstrate added diagnostic, prognostic, or treatment-selection value. Specialized cytokine testing should not be introduced for routine risk stratification on the basis of these findings. Future multicenter studies should record exact admission, medication, and sampling times; incorporate infection testing, age-appropriate blood-count interpretation, and lot-specific analytical limits; and compare acute, stable, and recovery states against clinically relevant outcomes.

Several limitations constrain inference. Convenience sampling at one hospital, 59 participants, only four severe cases, and the absence of non-asthmatic and stable-asthma comparison groups limit generalizability and specificity. Medication histories were not collected systematically, leaving pretreatment confounding unresolved. There was no microbiological workup. Revision-stage analyses relied on aggregate tables; continuous-cytokine analyses and reclassification using alternative age-specific cutoffs were not possible from these data. The subgroup comparisons added during revision were not prespecified in the original protocol and were explicitly included in the 46-test family. Crude ORs and unadjusted log-Wald intervals are descriptive, sometimes unstable, and not causal estimates. The cross-sectional design cannot assess future attacks, treatment response, or changes during recovery.

## 5. Conclusions

Study-defined eosinophilia and leukocytosis were common in these 59 hospitalized Vietnamese children, and IL-6 most frequently met its prespecified cytokine threshold. No clinical or cytokine association remained statistically significant after multiplicity adjustment, and the exact analysis did not support an eosinophilia–severity association. These findings describe peripheral inflammatory patterns but do not validate airway phenotypes, prognostic stratification, or treatment selection. Longitudinal studies with standardized treatment histories, sampling times, infection assessment, and analytical documentation are needed.

## Figures and Tables

**Table 1:** Clinical characteristics and availability of relevant covariates (*n* = 59).

Characteristic	Category/Documentation	Value
Age	Years, mean ± SD	7.75 ± 2.7
Sex	Male	42 (71.2)
Sex	Female	17 (28.8)
Asthma diagnosis	Previously diagnosed	45 (76.3)
Asthma diagnosis	First diagnosed at enrollment	14 (23.7)
Age at first diagnosis	<2 years	5 (8.5)
Age at first diagnosis	2–5 years	34 (57.6)
Age at first diagnosis	>5 years	20 (33.9)
Protocol BMI z-score category	<−2	4 (6.8)
Protocol BMI z-score category	−2 to +2	36 (61.0)
Protocol BMI z-score category	>+2	19 (32.2)
Personal allergic history	Yes	37 (62.7)
Family asthma/allergy history	Yes	29 (49.2)
Household tobacco smoke	Exposed	21 (35.6)
Exacerbation severity	Mild	22 (37.3)
Exacerbation severity	Moderate	33 (55.9)
Exacerbation severity	Severe	4 (6.8)
Blood collection timing	Immediately following admission	Exact interval unavailable
Pre-sampling asthma medication	Dose, route, and timing	Not systematically collected
Microbiological testing	Including *M. pneumoniae*	Not performed

Values are *n* (%) unless stated otherwise. SD, standard deviation; BMI, body mass index. “Not systematically collected” does not mean no treatment. Exact patient-specific admission-to-draw intervals were unavailable for analysis.

**Table 2:** Frequencies at or above the prespecified study thresholds.

Marker	Operational Threshold	At/Above Threshold, *n*/*N* (%)
Total white blood cells	≥10,000 cells/µL	42/59 (71.2)
Neutrophils	≥7000 cells/µL	32/59 (54.2)
Eosinophils	≥300 cells/µL	37/59 (62.7)
Basophils	≥50 cells/µL	13/59 (22.0)
IL-4	≥10.62 pg/mL	0/59 (0.0)
IL-5	≥6.836 pg/mL	14/59 (23.7)
IL-12p70	≥7.642 pg/mL	0/59 (0.0)
TNF-α	≥6.152 pg/mL	2/59 (3.4)
IFN-γ	≥14.233 pg/mL	0/59 (0.0)
IL-6	≥9.473 pg/mL	41/59 (69.5)

All denominators are 59. Cytokine cutoffs are not presented as independently verified LODs, LLOQs, or clinical reference limits. IL, interleukin; IFN, interferon; TNF, tumor necrosis factor.

**Table 3:** Clinical characteristics by blood eosinophil stratum.

Characteristic/Level	<300 (*n* = 22)	≥300 (*n* = 37)	Crude OR (95% CI)	Raw *p*	Holm *p*
Male sex (vs female)	18 (81.8)	24 (64.9)	0.41 (0.11–1.47)	0.164 ^a^	1.000
First diagnosis: <2 y	2 (9.1)	3 (8.1)	1 (reference)	0.777 ^c^	1.000
First diagnosis: 2–5 y	14 (63.6)	20 (54.1)	0.95 (0.14–6.46)	—	—
First diagnosis: >5 y	6 (27.3)	14 (37.8)	1.56 (0.20–11.83)	—	—
BMI z-score <−2	2 (9.1)	2 (5.4)	0.57 (0.07–4.50)	0.915 ^c^	1.000
BMI z-score −2 to +2	13 (59.1)	23 (62.2)	1 (reference)	—	—
BMI z-score >+2	7 (31.8)	12 (32.4)	0.97 (0.31–3.07)	—	—
Personal allergy: yes	9 (40.9)	28 (75.7)	4.49 (1.45–13.97)	0.008 ^a^	0.348
Family history: yes	6 (27.3)	23 (62.2)	4.38 (1.39–13.83)	0.010 ^a^	0.429
Smoke exposure: yes	8 (36.4)	13 (35.1)	0.95 (0.32–2.85)	0.924 ^a^	1.000
Severity: mild	8 (36.4)	14 (37.8)	1 (reference)	0.368 ^c^	1.000
Severity: moderate	14 (63.6)	19 (51.4)	0.78 (0.26–2.35)	—	—
Severity: severe	0 (0.0)	4 (10.8)	NE	—	—

Values are *n* (%) within stratum. Reference categories: female; first diagnosis <2 years; BMI z-score −2 to +2; no history/exposure; mild exacerbation. ORs describe eosinophilia; *p*-values are global. ^a^ Pearson χ^2^; ^c^ Fisher–Freeman–Halton exact. NE, finite positive OR/log-Wald CI not reported because of a zero cell. CIs are not multiplicity-adjusted.

**Table 4:** Cytokine study-threshold status by blood eosinophil stratum.

Cytokine	<300 (*n* = 22)	≥300 (*n* = 37)	Crude OR (95% CI)	Raw *p*	Holm *p*
IL-4	0 (0.0)	0 (0.0)	NA	NA	NA
IL-5	7 (31.8)	7 (18.9)	0.50 (0.15–1.69)	0.260 ^a^	1.000
IL-12p70	0 (0.0)	0 (0.0)	NA	NA	NA
TNF-α	0 (0.0)	2 (5.4)	NE	0.524 ^b^	1.000
IFN-γ	0 (0.0)	0 (0.0)	NA	NA	NA
IL-6	14 (63.6)	27 (73.0)	1.54 (0.50–4.78)	0.451 ^a^	1.000

Values are *n* (%) meeting the marker-specific threshold in Table 2. ORs compare the odds of meeting the cytokine threshold in the ≥300 group with the <300 group. ^a^ Pearson χ^2^; ^b^ two-sided Fisher exact. NA: no test or OR because both groups contained zero threshold-positive observations. NE: no finite log-Wald estimate owing to a zero cell. CIs are unadjusted for multiplicity.

## Data Availability

The data presented in this study are available from the corresponding author on reasonable request, subject to ethical and institutional restrictions related to pediatric clinical data.

## Supplementary Materials

The following supporting information can be downloaded at: https://www.mdpi.com/article/10.3390/pediatric18050135/s1. Table S1: Personal allergic history and study-threshold biomarkers; Table S2. Family history of asthma/allergy and study-threshold biomarkers; Table S3. Household tobacco smoke exposure and study-threshold biomarkers; Table S4. Exacerbation severity and study-threshold biomarkers; Table S5. Age at first asthma diagnosis and study-threshold biomarkers; Table S6. Protocol BMI category and study-threshold biomarkers; Table S7. Additional, nonduplicated eosinophil-stratum comparisons.
