# Supplementary material for: Peripheral Immune Cell Profiles and Circulating Cytokine Signals in Vietnamese Children Hospitalized with Acute Asthma Exacerbations: A Cross-Sectional Study
Source: Pediatr Rep. 2026 Oct 9;18(5):135. doi: 10.3390/pediatric18050135 (PMC13708134; doi:10.3390/pediatric18050135)
Supplement: Supplementary file 1 [file pediatrrep-18-00135-s001.zip › pediatrrep-4569318-supplementary.pdf]

Supplementary Table S1. Personal allergic history and study-threshold biomarkers

Biomarker	Factor category	At / above threshold n/N (%)	Crude OR (95% CI)	Raw p	Test	Holm p
Total white blood cells	Yes	28/37 (75.7)	1.78 (0.56–5.61)	0.323	Pearson	1.000
	No	14/22 (63.6)	1 (reference)			
Neutrophils	Yes	18/37 (48.6)	0.54 (0.18–1.60)	0.264	Pearson	1.000
	No	14/22 (63.6)	1 (reference)			
Eosinophils	Yes	28/37 (75.7)	4.49 (1.45–13.97)	0.008	Pearson	0.348
	No	9/22 (40.9)	1 (reference)			
Basophils	Yes	11/37 (29.7)	4.23 (0.84–21.28)	0.104	Fisher	1.000
	No	2/22 (9.1)	1 (reference)			
IL-5	Yes	7/37 (18.9)	0.50 (0.15–1.69)	0.260	Pearson	1.000
	No	7/22 (31.8)	1 (reference)			
TNF-α	Yes	2/37 (5.4)	NE	0.524	Fisher	1.000
	No	0/22 (0.0)	1 (reference)			
IL-6	Yes	24/37 (64.9)	0.54 (0.16–1.81)	0.317	Pearson	1.000
	No	17/22 (77.3)	1 (reference)			

Thresholds are defined in main-text Table 2. The p-value is the global factor × biomarker test; reference categories are shown explicitly. ORs are sample cross-product ratios with 95% log-Wald CIs; CIs are not multiplicity-adjusted. NE: no finite positive OR with a log-Wald CI reported owing to a zero cell. Exact tests use exhaustive fixed-margin probability ordering, not Monte Carlo sampling. Holm adjustment uses all 46 unique hypotheses, not a separate family for each table.

Supplementary Table S2. Family history of asthma/allergy and study-threshold biomarkers

Biomarker	Factor category	At / above threshold n/N (%)	Crude OR (95% CI)	Raw p	Test	Holm p
Total white blood cells	Yes	23/29 (79.3)	2.22 (0.69–7.12)	0.176	Pearson	1.000
	No	19/30 (63.3)	1 (reference)			
Neutrophils	Yes	17/29 (58.6)	1.42 (0.51–3.96)	0.506	Pearson	1.000
	No	15/30 (50.0)	1 (reference)			
Eosinophils	Yes	23/29 (79.3)	4.38 (1.39–13.83)	0.010	Pearson	0.429
	No	14/30 (46.7)	1 (reference)			
Basophils	Yes	8/29 (27.6)	1.90 (0.54–6.71)	0.312	Pearson	1.000
	No	5/30 (16.7)	1 (reference)			
IL-5	Yes	8/29 (27.6)	1.52 (0.45–5.11)	0.493	Pearson	1.000
	No	6/30 (20.0)	1 (reference)			
TNF-α	Yes	2/29 (6.9)	NE	0.237	Fisher	1.000
	No	0/30 (0.0)	1 (reference)			
IL-6	Yes	22/29 (75.9)	1.82 (0.59–5.63)	0.296	Pearson	1.000
	No	19/30 (63.3)	1 (reference)			

Thresholds are defined in main-text Table 2. The p-value is the global factor × biomarker test; reference categories are shown explicitly. ORs are sample cross-product ratios with 95% log-Wald CIs; CIs are not multiplicity-adjusted. NE: no finite positive OR with a log-Wald CI reported owing to a zero cell. Exact tests use exhaustive fixed-margin probability ordering, not Monte Carlo sampling. Holm adjustment uses all 46 unique hypotheses, not a separate family for each table.

Supplementary Table S3. Household tobacco smoke exposure and study-threshold biomarkers

Biomarker	Factor category	At / above threshold n/N (%)	Crude OR (95% CI)	Raw p	Test	Holm p
Total white blood cells	Yes	16/21 (76.2)	1.48 (0.44–4.98)	0.528	Pearson	1.000
	No	26/38 (68.4)	1 (reference)			
Neutrophils	Yes	10/21 (47.6)	0.66 (0.23–1.93)	0.448	Pearson	1.000
	No	22/38 (57.9)	1 (reference)			
Eosinophils	Yes	13/21 (61.9)	0.95 (0.32–2.85)	0.924	Pearson	1.000
	No	24/38 (63.2)	1 (reference)			
Basophils	Yes	5/21 (23.8)	1.17 (0.33–4.18)	1.000	Fisher	1.000
	No	8/38 (21.1)	1 (reference)			
IL-5	Yes	6/21 (28.6)	1.50 (0.44–5.11)	0.538	Fisher	1.000
	No	8/38 (21.1)	1 (reference)			
TNF-α	Yes	0/21 (0.0)	NE	0.534	Fisher	1.000
	No	2/38 (5.3)	1 (reference)			
IL-6	Yes	15/21 (71.4)	1.15 (0.36–3.71)	0.810	Pearson	1.000
	No	26/38 (68.4)	1 (reference)			

Thresholds are defined in main-text Table 2. The p-value is the global factor × biomarker test; reference categories are shown explicitly. ORs are sample cross-product ratios with 95% log-Wald CIs; CIs are not multiplicity-adjusted. NE: no finite positive OR with a log-Wald CI reported owing to a zero cell. Exact tests use exhaustive fixed-margin probability ordering, not Monte Carlo sampling. Holm adjustment uses all 46 unique hypotheses, not a separate family for each table.

Supplementary Table S4. Exacerbation severity and study-threshold biomarkers

Biomarker	Factor category	At / above threshold n/N (%)	Crude OR (95% CI)	Raw p	Test	Holm p
Total white blood cells	Mild	15/22 (68.2)	1 (reference)	0.606	Fisher–Freeman–Halton	1.000
	Moderate	23/33 (69.7)	1.07 (0.33–3.44)			
	Severe	4/4 (100.0)	NE			
Neutrophils	Mild	11/22 (50.0)	1 (reference)	0.711	Fisher–Freeman–Halton	1.000
	Moderate	18/33 (54.5)	1.20 (0.41–3.54)			
	Severe	3/4 (75.0)	3.00 (0.27–33.49)			
Eosinophils	Mild	14/22 (63.6)	1 (reference)	0.368	Fisher–Freeman–Halton	1.000
	Moderate	19/33 (57.6)	0.78 (0.26–2.35)			
	Severe	4/4 (100.0)	NE			
Basophils	Mild	7/22 (31.8)	1 (reference)	0.273	Fisher–Freeman–Halton	1.000
	Moderate	5/33 (15.2)	0.38 (0.10–1.42)			
	Severe	1/4 (25.0)	0.71 (0.06–8.15)			
IL-5	Mild	4/22 (18.2)	1 (reference)	0.794	Fisher–Freeman–Halton	1.000
	Moderate	9/33 (27.3)	1.69 (0.45–6.36)			
	Severe	1/4 (25.0)	1.50 (0.12–18.44)			
TNF-α	Mild	1/22 (4.5)	1 (reference)	1.000	Fisher–Freeman–Halton	1.000
	Moderate	1/33 (3.0)	0.66 (0.04–11.08)			
	Severe	0/4 (0.0)	NE			
IL-6	Mild	12/22 (54.5)	1 (reference)	0.145	Fisher–Freeman–Halton	1.000
	Moderate	26/33 (78.8)	3.10 (0.95–10.11)			
	Severe	3/4 (75.0)	2.50 (0.22–27.94)			

Thresholds are defined in main-text Table 2. The p-value is the global factor × biomarker test; reference categories are shown explicitly. ORs are sample cross-product ratios with 95% log-Wald CIs; CIs are not multiplicity-adjusted. NE: no finite positive OR with a log-Wald CI reported owing to a zero cell. Exact tests use exhaustive fixed-margin probability ordering, not Monte Carlo sampling. Holm adjustment uses all 46 unique hypotheses, not a separate family for each table.

Supplementary Table S5. Age at first asthma diagnosis and study-threshold biomarkers

Biomarker	Factor category	At / above threshold n/N (%)	Crude OR (95% CI)	Raw p	Test	Holm p
Total white blood cells	<2	4/5 (80.0)	1 (reference)	0.829	Fisher–Freeman–Halton	1.000
	2–5	25/34 (73.5)	0.69 (0.07–7.07)			
	>5	13/20 (65.0)	0.46 (0.04–5.00)			
Neutrophils	<2	2/5 (40.0)	1 (reference)	0.728	Fisher–Freeman–Halton	1.000
	2–5	18/34 (52.9)	1.69 (0.25–11.42)			
	>5	12/20 (60.0)	2.25 (0.30–16.63)			
Eosinophils	<2	3/5 (60.0)	1 (reference)	0.777	Fisher–Freeman–Halton	1.000
	2–5	20/34 (58.8)	0.95 (0.14–6.46)			
	>5	14/20 (70.0)	1.56 (0.20–11.83)			
Basophils	<2	1/5 (20.0)	1 (reference)	1.000	Fisher–Freeman–Halton	1.000
	2–5	8/34 (23.5)	1.23 (0.12–12.65)			
	>5	4/20 (20.0)	1.00 (0.09–11.59)			
IL-5	<2	2/5 (40.0)	1 (reference)	0.328	Fisher–Freeman–Halton	1.000
	2–5	6/34 (17.6)	0.32 (0.04–2.36)			
	>5	6/20 (30.0)	0.64 (0.08–4.89)			
TNF-α	<2	1/5 (20.0)	1 (reference)	0.064	Fisher–Freeman–Halton	1.000
	2–5	0/34 (0.0)	NE			
	>5	1/20 (5.0)	0.21 (0.01–4.12)			
IL-6	<2	4/5 (80.0)	1 (reference)	1.000	Fisher–Freeman–Halton	1.000
	2–5	23/34 (67.6)	0.52 (0.05–5.25)			
	>5	14/20 (70.0)	0.58 (0.05–6.37)			

Thresholds are defined in main-text Table 2. The p-value is the global factor × biomarker test; reference categories are shown explicitly. ORs are sample cross-product ratios with 95% log-Wald CIs; CIs are not multiplicity-adjusted. NE: no finite positive OR with a log-Wald CI reported owing to a zero cell. Exact tests use exhaustive fixed-margin probability ordering, not Monte Carlo sampling. Holm adjustment uses all 46 unique hypotheses, not a separate family for each table.

Supplementary Table S6. Protocol BMI category and study-threshold biomarkers

Biomarker	Factor category	At / above threshold n/N (%)	Crude OR (95% CI)	Raw p	Test	Holm p
Total white blood cells	z-score <−2	3/4 (75.0)	1.15 (0.11–12.44)	0.899	Fisher–Freeman–Halton	1.000
	z-score −2 to +2	26/36 (72.2)	1 (reference)			
	z-score >+2	13/19 (68.4)	0.83 (0.25–2.80)			
Neutrophils	z-score <−2	2/4 (50.0)	0.89 (0.11–7.06)	0.916	Fisher–Freeman–Halton	1.000
	z-score −2 to +2	19/36 (52.8)	1 (reference)			
	z-score >+2	11/19 (57.9)	1.23 (0.40–3.78)			
Eosinophils	z-score <−2	2/4 (50.0)	0.57 (0.07–4.50)	0.915	Fisher–Freeman–Halton	1.000
	z-score −2 to +2	23/36 (63.9)	1 (reference)			
	z-score >+2	12/19 (63.2)	0.97 (0.31–3.07)			
Basophils	z-score <−2	1/4 (25.0)	1.67 (0.15–18.87)	0.404	Fisher–Freeman–Halton	1.000
	z-score −2 to +2	6/36 (16.7)	1 (reference)			
	z-score >+2	6/19 (31.6)	2.31 (0.63–8.51)			
IL-5	z-score <−2	2/4 (50.0)	3.50 (0.42–28.91)	0.424	Fisher–Freeman–Halton	1.000
	z-score −2 to +2	8/36 (22.2)	1 (reference)			
	z-score >+2	4/19 (21.1)	0.93 (0.24–3.62)			
TNF-α	z-score <−2	0/4 (0.0)	NE	1.000	Fisher–Freeman–Halton	1.000
	z-score −2 to +2	1/36 (2.8)	1 (reference)			
	z-score >+2	1/19 (5.3)	1.94 (0.11–32.93)			
IL-6	z-score <−2	4/4 (100.0)	NE	0.273	Fisher–Freeman–Halton	1.000
	z-score −2 to +2	26/36 (72.2)	1 (reference)			
	z-score >+2	11/19 (57.9)	0.53 (0.16–1.70)			

Thresholds are defined in main-text Table 2. The p-value is the global factor × biomarker test; reference categories are shown explicitly. ORs are sample cross-product ratios with 95% log-Wald CIs; CIs are not multiplicity-adjusted. NE: no finite positive OR with a log-Wald CI reported owing to a zero cell. Exact tests use exhaustive fixed-margin probability ordering, not Monte Carlo sampling. Holm adjustment uses all 46 unique hypotheses, not a separate family for each table.

Supplementary Table S7. Additional, nonduplicated eosinophil-stratum comparisons

Characteristic	Eosinophil stratum	Characteristic present n/N (%)	Crude OR (95% CI)	Raw p	Test	Holm p
Male sex	≥300 cells/μL	24/37 (64.9)	0.41 (0.11–1.47)	0.164	Pearson	1.000
	<300 cells/μL	18/22 (81.8)	1 (reference)			
IL-5	≥300 cells/μL	7/37 (18.9)	0.50 (0.15–1.69)	0.260	Pearson	1.000
	<300 cells/μL	7/22 (31.8)	1 (reference)			
TNF-α	≥300 cells/μL	2/37 (5.4)	NE	0.524	Fisher	1.000
	<300 cells/μL	0/22 (0.0)	1 (reference)			
IL-6	≥300 cells/μL	27/37 (73.0)	1.54 (0.50–4.78)	0.451	Pearson	1.000
	<300 cells/μL	14/22 (63.6)	1 (reference)			

For cytokines, "present" means at or above the study threshold, not analytically detectable. ORs compare the odds of the listed characteristic in the ≥300 group with the <300 group. The sex OR is algebraically identical to the male-versus-female OR for eosinophilia in main-text Table 3. Clinical hypotheses already included in Tables S1–S6 are not counted again. No tests were performed for IL-4, IL-12p70, or IFN-γ because all samples were below their study thresholds.

Reproducibility and scope

The 42 original hypotheses comprise six factors × seven varying biomarkers; the four additional comparisons in Table S7 increase the family to 46. Holm adjustment was applied to unrounded p-values [main-text reference 17]. The analyses use aggregate counts and do not reconstruct or impute individual participant data.

Statistical formulas: $OR = ad/bc$; 95% CI = $\exp[\ln(OR) \pm 1.959964 \times \sqrt{(1/a + 1/b + 1/c + 1/d)}]$. For group sizes n_i, positive counts x_i, and total positives K, the fixed-margin table probability is $\prod C(n_i, x_i) / C(\Sigma n_i, K)$. The two-sided exact p-value sums probabilities no greater than that of the observed table.
